# A simple and cost-effective setup for super-resolution localization microscopy

**DOI:** 10.1038/s41598-017-01606-6

**Published:** 2017-05-08

**Authors:** Hongqiang Ma, Rao Fu, Jianquan Xu, Yang Liu

**Affiliations:** 1 Biomedical and Optical Imaging Laboratory, Departments of Medicine and Bioengineering, University of Pittsburgh, Pittsburgh, PA 15213 USA; 2 College of Chemical Engineering, Northeast Electric Power University, Jilin Province, China; 3 University of Pittsburgh Cancer Institute, Pittsburgh, PA 15213 USA

## Abstract

Single molecule localization microscopy (SMLM) has become a powerful imaging tool for biomedical research, but it is mostly available in imaging facilities and a small number of laboratories due to its high cost. Here, we evaluate the possibility of replacing high-cost components on standard SMLM with appropriate low-cost alternatives and build a simple but high-performance super-resolution SMLM setup. Through numerical simulation and biological experiments, we demonstrate that our low-cost SMLM setup can yield similar localization precision and spatial resolution compared to the standard SMLM equipped with state-of-the-art components, but at a small fraction of their cost. Our low-cost SMLM setup can potentially serve as a routine laboratory microscope with high-performance super-resolution imaging capability.

## Introduction

In the past few decades, fluorescence microscopy has significantly expanded our ability to study biological processes at the cellular and subcellular level. However, due to the diffraction-limited spatial resolution, conventional fluorescence microscopy cannot visualize biological structures smaller than ~200 nm. Until the recent decade, a number of super-resolution imaging techniques have been developed to break the diffraction barrier. These super-resolution techniques can generally be divided into two categories: the technique based on the nonlinear effects of the fluorescence that sharpens the point spread function (PSF) using modulated light, including stimulated emission depletion (STED)^1^ and structured-illumination microscopy (SIM)^2, 3^; and the technique based on localization of sparsely excited single fluorescent emitters, including (fluorescence) photo-activated localization microscopy [(f) PALM]^4, 5^ and (direct) stochastic optical reconstruction microscopy [(d) STORM]^6, 7^. These super-resolution techniques have improved the spatial resolution by approximately an order of magnitude. In particular, single molecule localization microscopy (SMLM), which provides impressive spatial resolution down to 20 nm with relatively simple hardware compared to SIM and STED, has a great potential as a common laboratory tool for biomedical research. However, despite that SMLM has been commercialized for almost a decade, it remains a high-end microscopy instrument only available in the imaging facilities at major academic institutions and a small number of laboratories. Its high cost limits its widespread use as a routine microscopy system as conventional fluorescence microscope.

The key technical requirement for SMLM includes (1) a powerful laser to photo-bleach most fluorophores and activate only a small portion of sparsely distributed single fluorophores; (2) high numerical aperture (NA) objective lens to efficiently collect the limited number of photons emitted by single molecule; and (3) scientific cameras with high quantum efficiency and low noise to record the image from individual fluorescent emitters at a high signal to noise ratio (SNR). To meet these technical requirements and achieve the best performance, it is a common belief that the high-end optical and optoelectronic components have to be used, including high-power single-mode laser, high-end objectives, nano-positioning mechanical system and highly sensitive cameras, which easily drive the total cost to exceed $100,000.

To lower the cost of light source (one of the most expensive items), the industry-grade laser was proposed to replace the scientific grade laser, which has shown a decent performance in SMLM^8, 9^. Unlike SIM and STED, the imaging quality of SMLM is not sensitive to the slight degradation on the stability of the laser output and spectral linewidth. In contrast, the imaging quality of SMLM is more likely to be affected by the objective lens and cameras. Low-cost objective lens usually have lower NA, which not only degrades the spatial resolution, but also lowers the photon collection efficiency. When further combined with a low-cost camera with a relatively poor SNR, the final spatial resolution of SMLM can be severely degraded^9, 10^.

In this paper, we investigate the influence of the three major high-cost components in SMLM (light source, objective and camera) and identify the most appropriate low-cost alternatives to build a high-quality SMLM setup with a total cost of only ~$4,000 (Table S1 to compare with the estimated cost of standard SMLM shown in Table S2). Through numerical simulation and experiments with nanoparticles and biological cells, we demonstrate that the proposed cost-efficient SMLM setup can achieve a localization precision close to that of a standard SMLM setup using high-end components. We expect that this cost-efficient SMLM setup may serve as a promising low-cost routine microscope that can be used in most laboratories to take advantage of the super-resolution imaging capability.

## Results

### Schematic of the low-cost SMLM system

**Figure 1:**
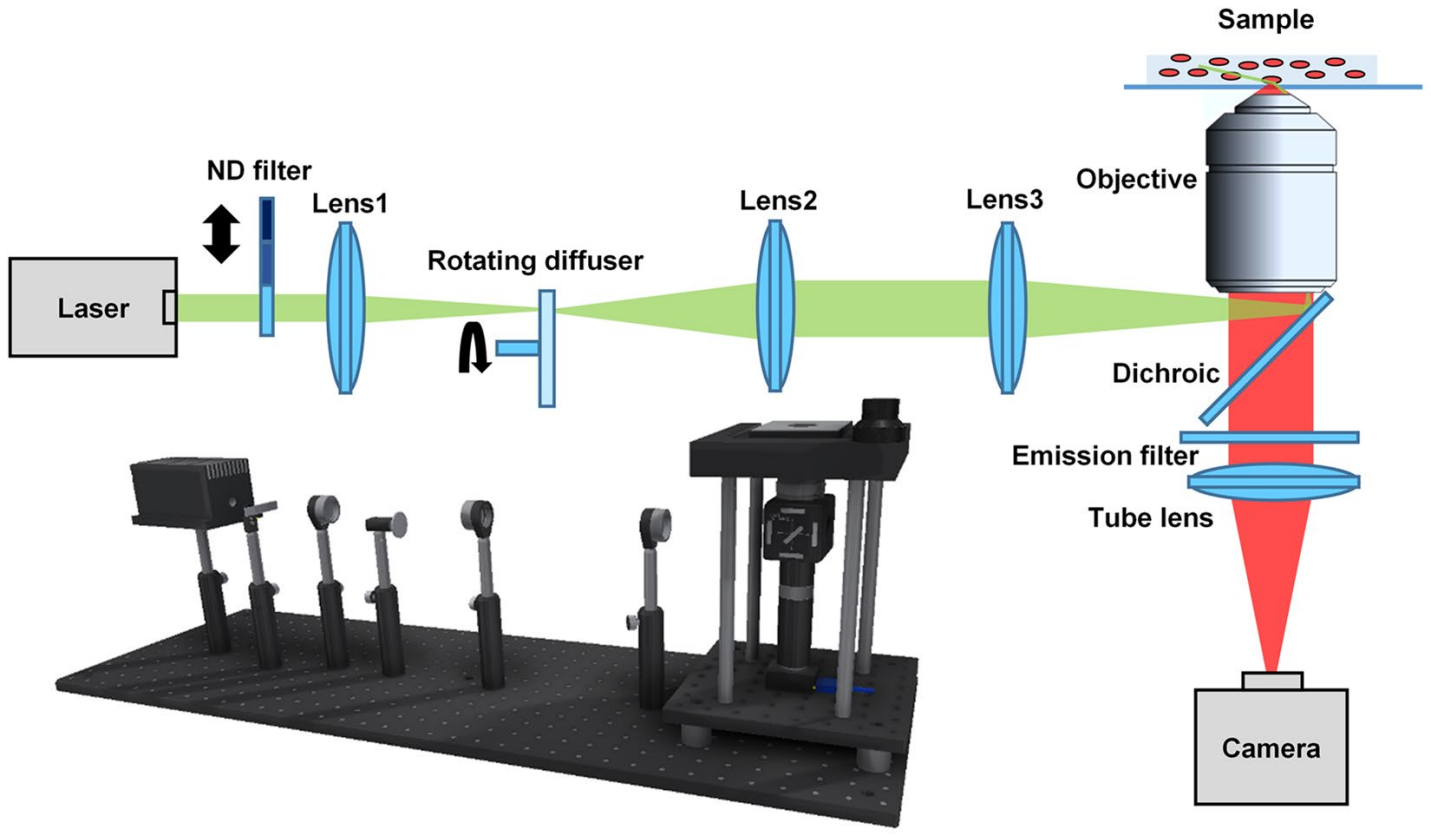
The schematic diagram of the low-cost SMLM setup. ND filter is a step variable metallic neutral density filters (NDL-10S-2, Thorlabs). Lens 1 and Lens 2 are achromatic lenses with a focal length of 100 mm (AC254-100-A, Thorlabs). Rotating diffuser is an engineered diffuser (EDC-5-06217-A, RPC Photonics) driven by a small DC motor (RB-Ada-142, RobotShop) with a rotation speed of 9100 rpm. Lens 3 is an achromatic lens with focal length of 200 mm (AC254-200-A, Thorlabs). The objective lens is a 100x oil immersion infinity-corrected Plan Fluor objective lens with a NA of 1.3. Dichroic mirror (FF660-Di02, Semrock) and emission filter (ET700/75 m, Chroma) are designed for the commonly used fluorophore, Alexa-647. The tube lens is an achromatic lens with focal length of 100 mm (AC254-100-A, Thorlabs), corresponding to an optical magnification of 60X. The camera (Chameleon3 CM3-U3-31S4M, PointGrey) is an industry CMOS camera without cooling, the sensor model is IMX265 with a pixel size of 3.45 µm, QE of 71% and read noise of 2.89 electrons. In this setup, 2 × 2 physical pixels are binned as one image pixels, corresponding to 115 nm per pixel in the sample plane.

A general schematic of our cost-effective SMLM system is shown in Fig. 1. The light source is an industry-grade diode laser with a central wavelength at 638 nm and a maximal output power of 2000 mW. The power of the laser beam is adjusted by the neutral-density (ND) filter, and then focused onto a rotating diffuser by Lens 1 to homogenize the spatial distribution of the laser beam and convert the beam profile to a flat-top shape. Next, the engineered flat-top laser beam is collimated by Lens 2 and projected onto the sample plane as a uniform illumination field by Lens 3 and objective lens. Upon illumination, the fluorophores emitted from the sample are collected by the objective lens, passing through the dichroic mirror, emission filter and are finally projected onto the camera sensor for image recording. The detailed list of suppliers and the cost for each component are provided in the Supplementary Material (Fig. S1 and Table S1). In this setup, three critical low-cost components that replace the high-end components in the standard SMLM are laser (~$409), objective ($549) and camera ($490). We next discuss the impact of these three components on the image quality and spatial resolution of SMLM.

### Illumination uniformity from the low-cost laser

The image quality and spatial resolution of SMLM are closely related to the uniform spatial distribution of illumination intensity across the imaging field of view (FOV). Heterogeneous illumination field can result in different background signal, different photon emission rate and thus different molecular density. Therefore, a flat-field illumination is important to SMLM imaging to ensure a high-quality super-resolution image with a uniform spatial resolution. To increase the uniformity across the FOV, standard refractive beam shaping optics can be used to convert the beam shape from Gaussian to flat-top with excellent performance, but it is expensive and only suitable for high-quality laser source. Alternatively, the most cost-effective approach is the engineered diffuser. However, optical diffuser suffers from two main drawbacks. First, it is often accompanied by laser speckle, which can be efficiently suppressed by averaging numerous illumination fields from a fast rotating diffuser as shown in Fig. 1; second, optical diffuser cannot be used in the configuration of total internal reflection fluorescence (TIRF) or highly inclined illumination, as it relies on regenerating numerous secondary point sources from the diffuser, resulting in a highly diverging beam that is often difficult to collimate. Here, we address this challenge by putting the diffuser at the focal plane of the focusing lens (Lens 1) and the collimating lens (Lens 2). The regenerated secondary point sources from the diffuser is highly confined in a small focal area at the focal point, and then after passing through Lens 2, a relatively collimated flat-top laser beam can be generated.

**Figure 2:**
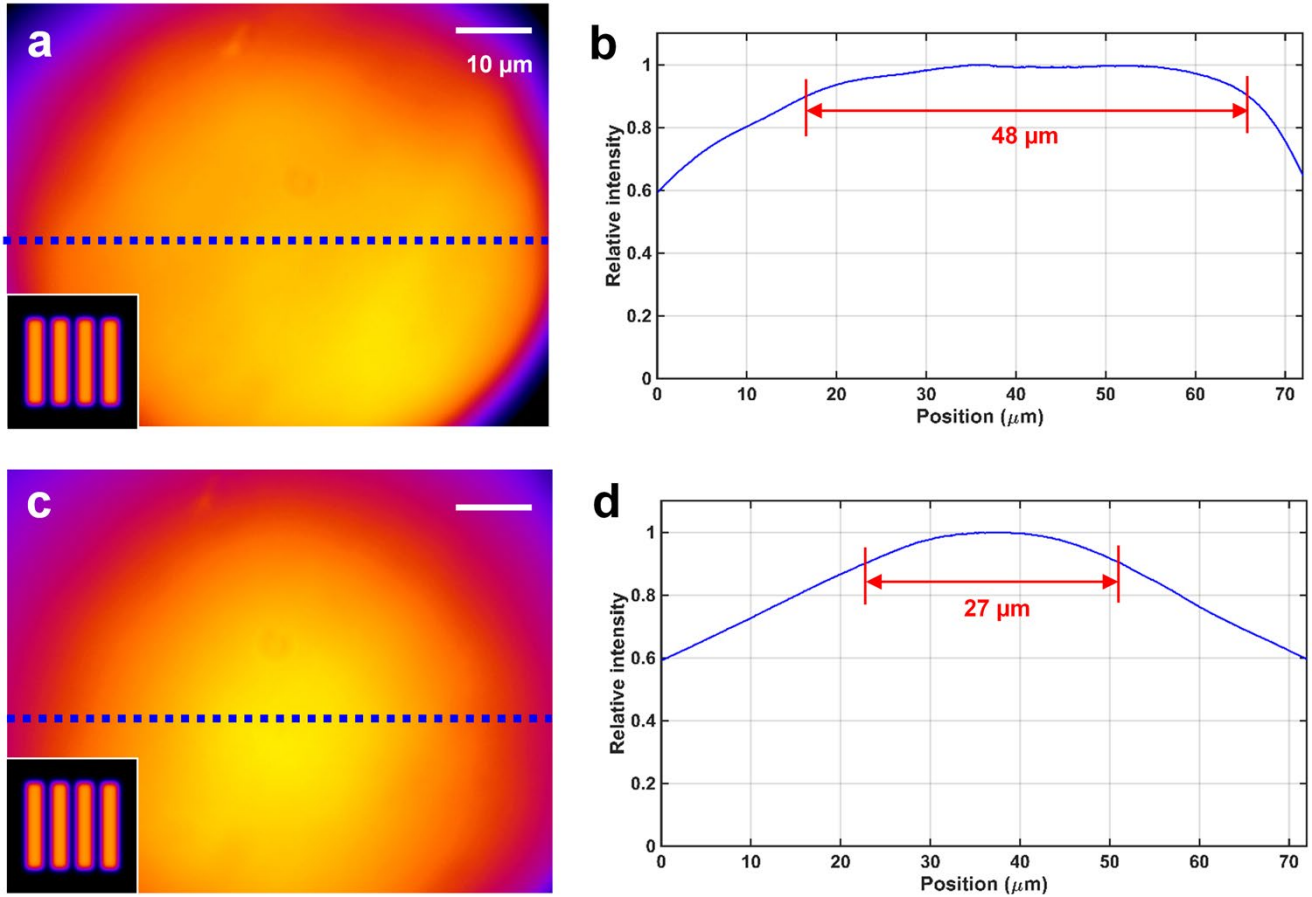
Uniformity of the illumination field in the low-cost SMLM by using different diffusers. (**a**,**c**) Spatial distribution of illumination intensity generated by the flat-top diffuser. (**b**) The corresponding intensity profile across the blue dotted line in a. The size of the illumination field with a relative intensity larger than 90% is 48 µm. (**c**) Spatial distribution of illumination intensity generated by the Gaussian diffuser (#47-994, Edmund Optics). (**d**) The corresponding intensity profile across the blue dotted line in (**c**). The size of the illumination field with a relative intensity larger than 90% is 27 µm. Note that, the inset shown in the left bottom corner in (**a**,**b**) is the original illumination pattern without diffuser.

To measure the uniformity of the illumination field, we obtained the fluorescent images from a highly concentrated fluorescent (Alexa-647) solution with a relatively low illumination intensity (~100 W/cm^2^). We found that our low-cost illumination strategy can efficiently produce a relatively homogeneous illumination field (Fig. 2(a,b)) with a large FOV (~48 µm). In comparison, we also tested the illumination profile generated with a Gaussian diffuser (Fig. 2(c,d)). Its illumination uniformity is also significantly better than that of the original illumination field (insets of Fig. 2(a,c)) without additional optimization, but is still not as homogeneous as that from the flat-top diffuser. Note that, the FOV of this setup can be easily enlarged to 117 × 88 µm^2^ (sensor size), if epi-illumination is used, rather than the highly inclined illumination used here (for lower background).

### Evaluation of imaging performance of low-cost objective and camera

Besides a low-cost laser, we also use a low-cost oil immersion objective with a NA of 1.3 to replace the commonly used the TIRF objective with a NA of 1.49, and an industry-grade CMOS camera to replace the commonly used EMCCD or sCMOS cameras in this low-cost SMLM setup.

**Figure 3:**
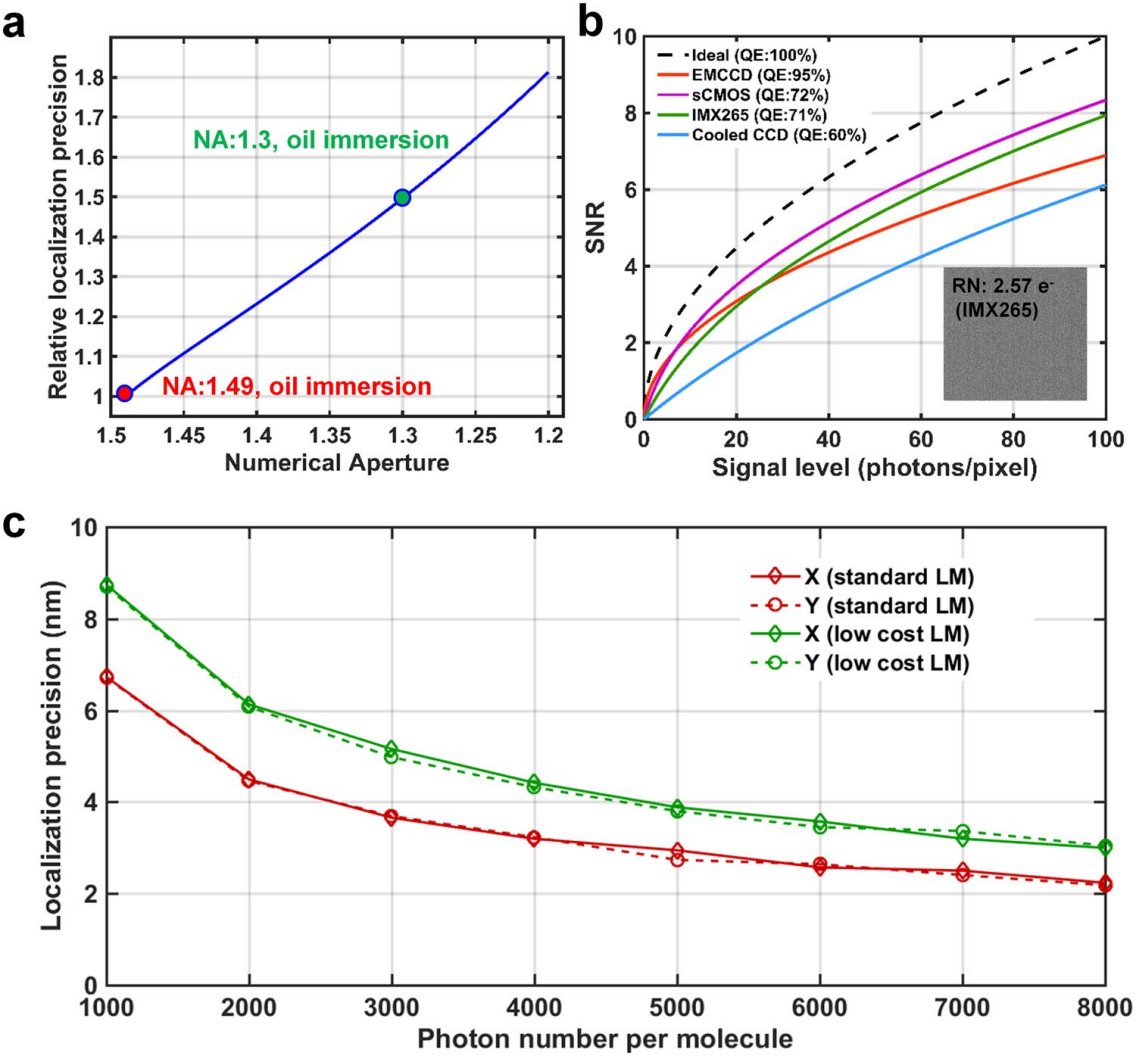
Comparison of localization performance by theoretical calculation and numerical simulation. (**a**) Comparison of relative localization precision for the objectives with different NAs. (**b**) Comparison of localization precision for different cameras under different signal levels. (**c**) Comparison of localization precision between the low-cost SMLM and the standard SMLM under different signal levels using simulated images. 1000 images were simulated for each data point. Here, the standard SMLM uses TIRF objective (100X, NA1.49) and EMCCD camera with a pixel size of 160 nm.

With regard to the objective, a lower NA often reduces the photon collection efficiency, enlarges the diffraction-limited point spread function (PSF), and thus degrades the localization precision, which is determined by $S/\sqrt{N}$, where *S* is the standard deviation of the PSF determined by the objective and *N* is the collected photons per molecule. Therefore, compared to the objective lens with a NA of 1.49, our low-cost objective theoretically leads to 50% degradation in localization precision and spatial resolution (Fig. 3a).

With regard to the camera, low cost usually means low QE and high noise. Therefore, standard SMLM prefers EMCCD or sCMOS cameras to achieve a high SNR. EMCCD camera has the advantage of high QE (~95%) and low read noise (<0.5 electron), but suffers from excess noise of $\sqrt{N}$, where N is the collected number of photons per pixel. In comparison, sCMOS camera has a relatively lower QE (~72%) and higher read noise (~1.6 electron), but without the influence of excess noise. Excess noise increases with the incident signal, and when the incident photons exceeds 7 photons per pixel, the SNR of the image recorded by the sCMOS camera would be superior to that of EMCCD cameras, as shown in Fig. 3b. Therefore, in the signal level of SMLM imaging (usually >10 photons per pixel), CMOS-based camera can potentially perform better. We identified a low-cost CMOS camera of IMX265 that has a QE of 71% (similar to that of sCMOS camera) and a read noise of 2.89 electrons (only slightly larger than that of sCMOS), which is expected to have a similar performance as that of some sCMOS cameras, and superior to EMCCD cameras when the signal level larger than 22 photons per pixel, as shown in Fig. 3b.

Finally, we evaluated the performance of this combination of low-cost objective and CMOS camera in localization precision with that of the standard SMLM equipped with TIRF objective (NA = 1.49) and EMCCD camera via a series of simulated images at different signal levels. Our numerical simulation shows that this low-cost combination achieves a localization precision very close to that of a standard SMLM using high-end components under a wide range of signal level. In particular, at the signal level close to the commonly used fluorophore Alexa-647 (~5000 photons per localization), the localization precision of this low-cost SMLM can reach 4 nm in the lateral dimension, only 1 nm larger than the state-of-the-art SMLM. Even at the signal level of PALM (~1000 photons per localization), this low-cost system still achieves a satisfactory localization precision that is better than 10 nm.

### Evaluation of the imaging performance via super-resolution imaging of nanoparticles

**Figure 4:**
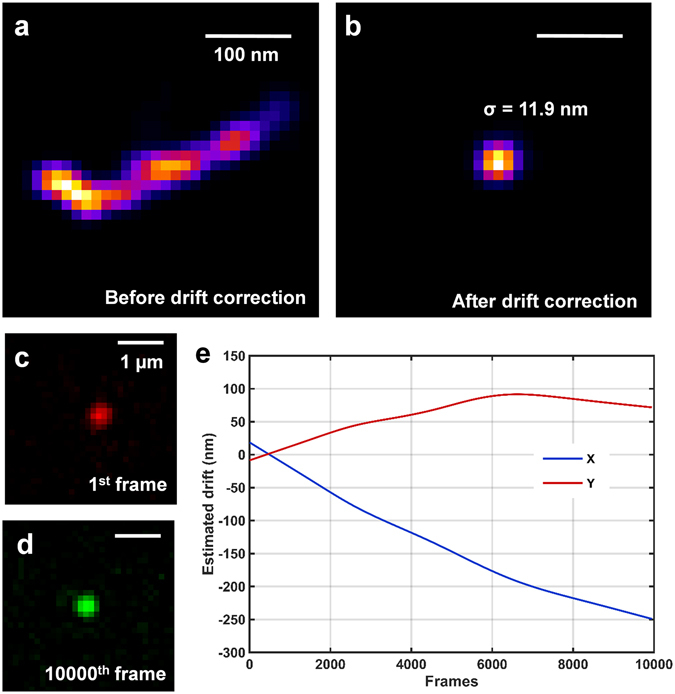
Performance of our low-cost SMLM for super-resolution imaging of nanoparticles. (**a**,**b**) Reconstructed super-resolution image of the nanoparticle (**a**) without drift correction and (**b**) with cross-correction based *posteriori* drift correction. Note that, the final localization precision of our low-cost SMLM setup is 11.9 nm. (**c**) The fluorescence image of the nanoparticle from the first image frame. (**d**) The fluorescence image of the same nanoparticle after ~7 minutes from 10000^th^ frame. The nanoparticle still stays at the focal plane without visible focus drift, suggesting that the effect of axial drift can be neglected for 2D imaging up to 10,000 frames. (**e**) The lateral sample drift for 10,000 frames estimated by the cross-correlation based drift correction algorithm integrated in ThunderSTORM.

Next, we evaluated the performance of our low-cost SMLM setup with an experiment using gold nanoparticles. We captured 10,000 frames when imaging the gold nanoparticle (100 nm diameter) at a frame rate of 25 fps with an illumination power density of ~10 kW/cm^2^ and an average photon number of ~8,000 per localization. Similar to the standard SMLM setup, our low-cost SMLM setup is also sensitive to sample drift due to the long acquisition time (Fig. 4a). However, the lateral drift can be well estimated by post processing with cross-correlation based algorithm^11^ (Fig. 4e), and it shows a localization precision of 11.9 nm in this experiment after *posteriori* drift correction (Fig. 4b). This precision is similar to that of a commercial STORM system^12^, but is still worse than the simulation results. A possible reason is that the cross-correlation based method cannot efficiently correct for high-frequency drift. If a higher localization precision is required, fiducial markers are needed for more accurate drift correction.

To evaluate the drift in the axial dimension, we compared the PSF pattern of the gold nanoparticle in the first and the last frame (Fig. 4c,d) and found the nanoparticle was kept at the image plane without obvious defocusing effect, suggesting that the effect of axial drift in this low-cost SMLM system can be neglected for 2D imaging up to 10,000 frames.

### Evaluation of super-resolution imaging performance via *d*STORM imaging of microtubules

**Figure 5:**
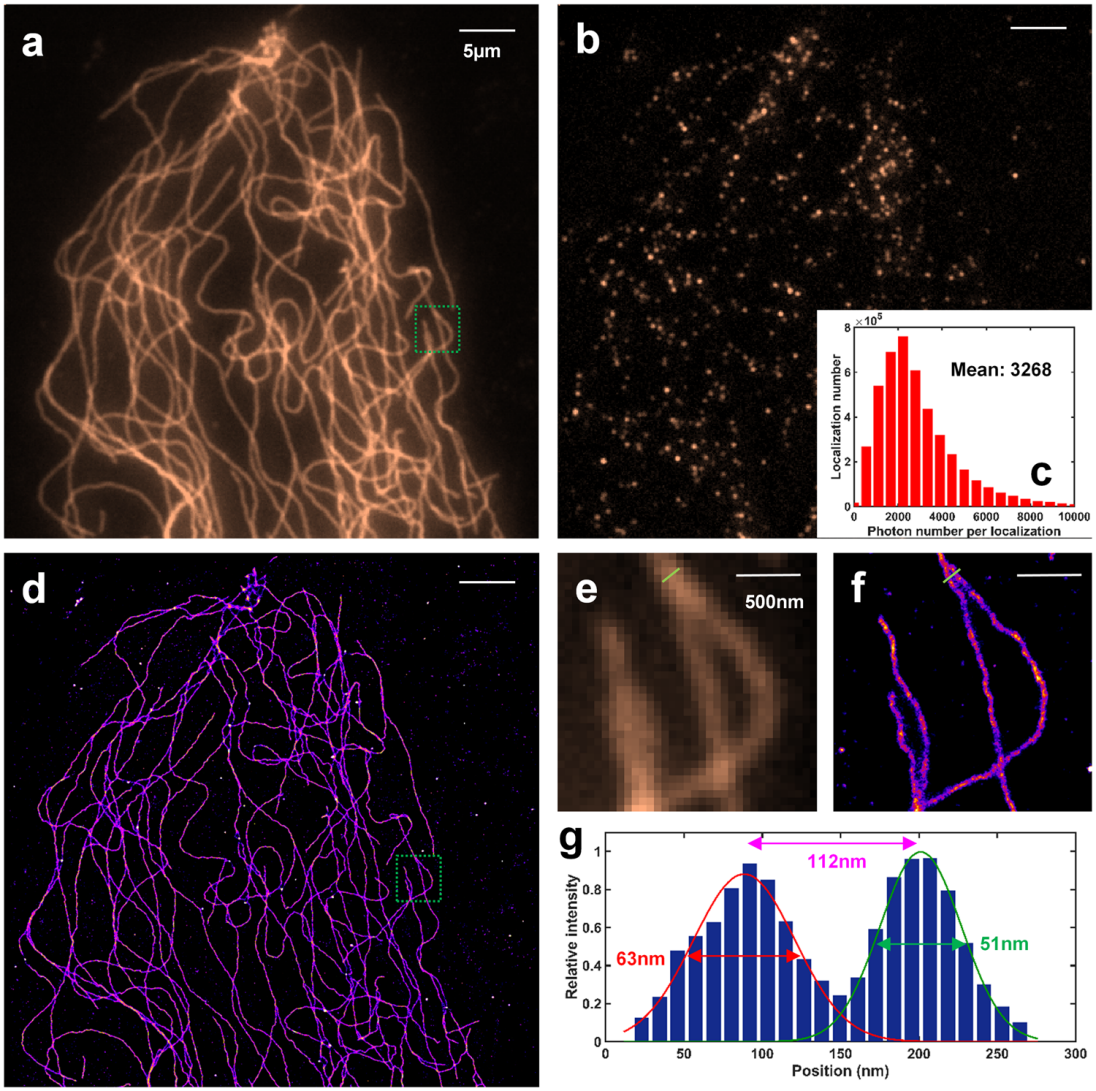
Performance of low-cost SMLM imaging of microtubules in MEF cells. (**a**) Conventional wide-field fluorescence image. (**b**) A single raw image under *d*STORM imaging condition. (**c**) Statistical distribution of the photon number per localization event in this experiment. (**d**) The reconstructed super-resolution image. Pixel size: 11.5 nm. (**e**,**f**) are the zoomed-in region of the green box in (**a**,**d**). (**g**) Statistical distribution of the localization events along the green line in (**f**).

Next, we evaluated the performance of our low-cost SMLM via imaging the commonly used biological sample of microtubules. The microtubules in fixed primary mouse embryo fibroblast (MEF) cells were labeled with Alexa-647 and imaged by our low-cost SMLM setup with an illumination power density of ~10 kW/cm^2^. To reconstruct the final super-resolution image, 10,000 frames were acquired at a frame rate of 25 fps and the super-resolution image was reconstructed by the widely used free software ThunderSTORM^11, 13^ based on ImageJ platform. As shown in Fig. 5, the microtubules in the reconstructed super-resolution image clearly exhibit finer structures compared to that from conventional wide-field fluorescence image. For quantitative comparison, we selected an area of two closely spaced microtubules as shown in Fig. 5(e,f), and quantified their molecular count distribution. We found that our SMLM setup yields a lateral full width at half maximum (FWHM) of 51 nm and 63 nm (Fig. 5g) for each microtubule (in good agreement with the previously published results^14^), and can clearly resolve two microtubules separated by 112 nm. Note that, the average photon number per localization event is 3268 in this experiment, which is also consistent with the previous characterization of photon number from Alexa 647.

### Evaluation of super-resolution imaging performance via *d*STORM imaging of histone clusters

**Figure 6:**
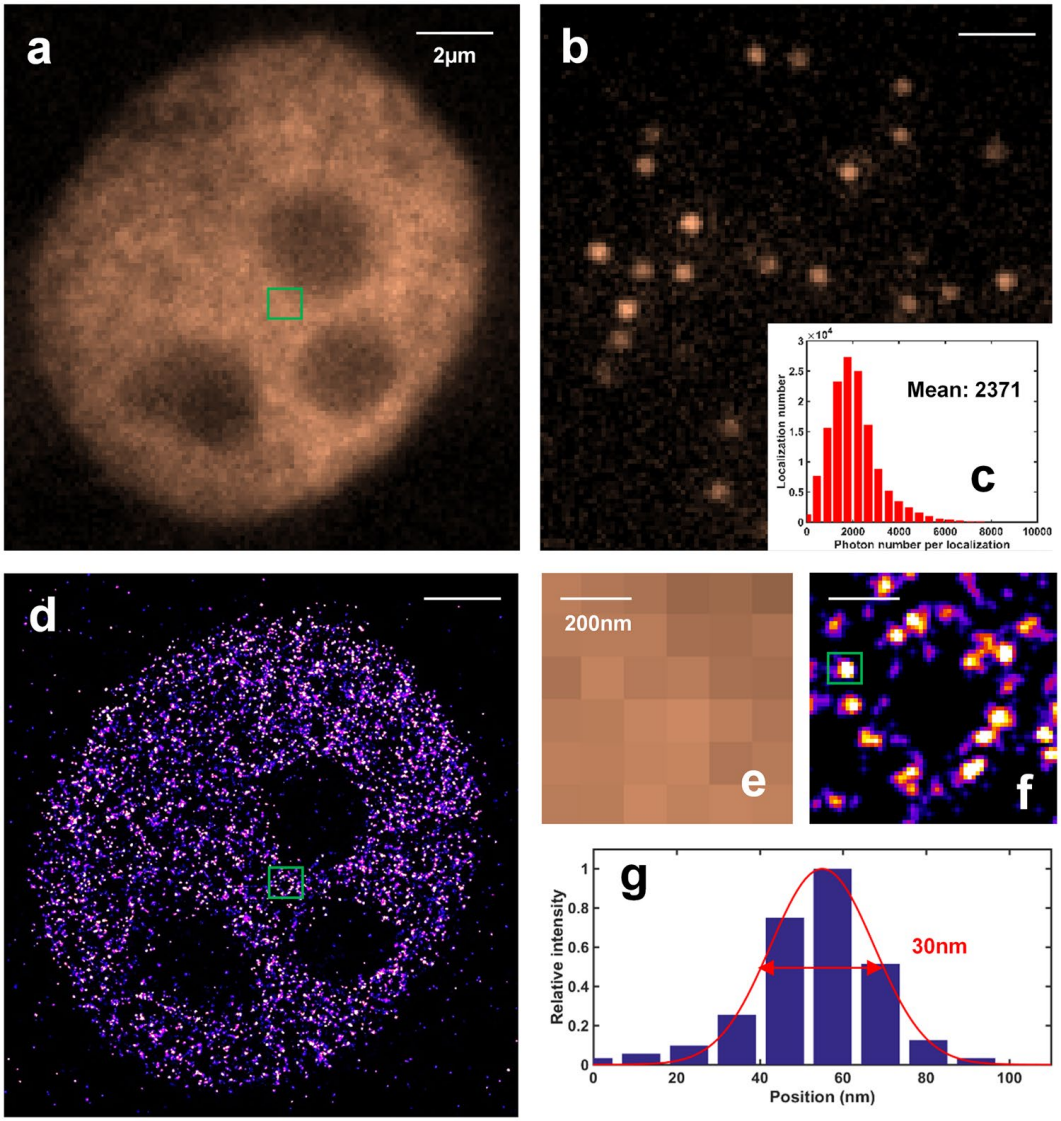
The performance of our low-cost SMLM imaging of acetylated H3 protein in MCF10A cells. (**a**) Conventional wide-field image. (**b**) A single raw image under *d*STORM imaging condition. (**c**) Statistical distribution of the photon number per localization event in this experiment. (**d**) The reconstructed super-resolution image. Pixel size: 11.5 nm. (**e**,**f**) are the zoomed-in region of the green box in (**a**,**d**). (**g**) Statistical distribution of the localization events for the selected acetylated H3 cluster in the green box of (**f**).

It is well-known that microtubules in MEF cells are one of the easiest structures to image by dSTORM, due to their well-defined and densely labeled structures and relatively shallow imaging depth. Next, we evaluated the performance of this low-cost SMLM when imaging a more challenging structure of nucleosomes in the cell nucleus^15^, due to the lower labeling density and thicker depth within the nucleus. The acetylated histone protein H3 was labeled with Alexa-647 in the fixed MCF10A cells, imaged with our low-cost SMLM setup and reconstructed by ThunderSTORM. Since the nucleus is located at a much deeper depth (a few microns above the surface of the coverslip) than that of microtubules, the aberration caused by the mismatch of refractive index between the immersion oil and imaging buffer is much more obvious. To reduce the refractive index mismatch, we introduce 60% 2,2′-thiodiethanol (TDE) to the imaging buffer^16, 17^, in which the aberration is significantly reduced without reducing the blinking properties of the fluorophore. The collected photon number reaches 2371 per localization event under this condition, which is 27% less than that of the previous experiment of imaging microtubules. Although there is a small loss in photon number, the resolution improvement allows the visualization of individual acetylated H3 histone clusters (Fig. 6d,f), which clearly are not visible in the corresponding wide-field images (Fig. 6a,e). We also quantified the size of acetylated H3 clusters, which shows a lateral FWHM of 30 nm, consistent with the previously published results^15^.

## Discussion

In this study, we present a simple and low-cost setup for SMLM imaging by proper integration of the cost-effective light source, objective and camera. It is generally believed that for super-resolution single molecule SMLM imaging at a resolution of less than 50 nm, high-end optics and optoelectronics devices have to be used. Although numerical simulation suggests that the low-cost SMLM results in a worse localization precision compared to the standard SMLM with high-end components, the difference is only a few nanometers which may be not significant in many biological experiments. Indeed, our experiments for imaging microtubules and nucleosomes show that this cost-effective SMLM setup can achieve a similar localization precision compared to the standard SMLM setup with high-end components. Our simple method to generate uniform illumination from low-cost laser diodes applies to epi-illumination, highly inclined illumination and total internal reflection fluorescence (TIRF) configuration (see Fig. S2). Moreover, in consideration of the high-NA objective is usually more sensitive to aberrations, photons from the edge from the objective pupil do not truly contribute to the main peak of the diffraction spot on the image plane, but form sidelobes that adds background^18^, so the actual photon collection efficiency from an objective with a NA of 1.49 may not be as high one would expect from theoretical calculation. Therefore, the performance of the low-cost objective lens with NA of 1.3 is in fact not as poor as one would expect. In addition, the performance of this low-cost SMLM system can be further improved, if IMX250 sensor is used. It is only slightly more expensive than IMX265 (by a few hundred dollars), but can yields 60% larger FOV, 120% higher throughput, 5% higher QE, and 0.52 electrons smaller read noise. Moreover, this low-cost setup can be easily extended for multi-color imaging and 3D imaging with multiple cameras to increase the throughput of current SMLM system. One drawback of this low-cost SMLM setup is the lack of online drift correction module. In our setup, we simply used the rubber feet to replace the high-end optical table to isolate the vibration. Our results (Fig. 4) show that by using vibration isolating feet, the effect of axial drift is minimal for 2D imaging up to 10,000 frames, and cross-correlation based image registration method^19^ can well compensate the long-term lateral drift. But for 3D imaging or 2D imaging for an extended time, the additional online correction components (e.g., nano-positioning stage) needs to be introduced to obtain the calibration curves and perform online drift correction during the image acquisition^12^. In conclusion, we present a high-quality low-cost SMLM setup with a comparable performance to that of standard SMLM. This work opens up the possibility of low-cost super-resolution SMLM imaging system as a routine laboratory microscope as conventional fluorescence or phase-contrast microscope, so that most biomedical research laboratories around the world can access the powerful super-resolution imaging capability.

## Materials and Methods

### SNR definition for camera comparison

The SNR is defined as follows:

$$\begin{array}{c}SNR=Signal\cdot QE/\sqrt{Signal\cdot QE+ExcessNoise\cdot Signal\cdot QE+ReadNois{e}^{2}}\\ \{\begin{array}{l}ExcessNoise=1,EMCCD\\ ExcessNoise=0,other\,cameras\end{array}\end{array}$$

Please note that, only quantum efficiency (QE) and read noise are considered. The effect of dark current noise is ignored. For camera without cooling, the dark current noise can be up to several electrons per pixel per second under the room temperature, which needs to be significantly reduced via deep cooling if long exposure time (e.g., several seconds) is required. But it is not crucial in the context of SMLM imaging, because the acquisition speed is usually several tens of frames per second; and for each frame, the dark current noise is far less than 1 electron, which can be neglected. To validate this point, we measured the sum of the read noise and dark current noise of IMX265 under the experimental condition^20^, and found it is only 2.57 electrons.

### Numerical simulation

To compare the performance of our low-cost SMLM setup with the standard SMLM setup, a series of image sets with different signal levels are numerically generated^21^. For each image, a single molecule is randomly distributed in the central pixel. The PSF was modeled with Gaussian function with a kernel width of 0.21 · *λ*/*NA*. The wavelength λ is set to be 700 nm to mimic the wavelength of a commonly used fluorophore (Alexa 647). The pixel size is set to be 160 nm for standard SMLM setup equipped with TIRF objective (100X oil, NA = 1.49) and EMCCD cameras (iXon 897, Andor) and 115 nm for our low-cost SMLM setup. The total photon number of the molecules is set to be from 1000 to 8000 to cover the signal range of the commonly used fluorescent dyes. The background for each pixel is set to be 1/50^th^ of total photon number per molecule. The noise is modeled with a Poisson model plus the excess noise for standard SMLM, and Poisson model plus the read noise with standard deviation of 2.57 electrons for our low-cost SMLM. For each signal level, 1000 images are generated and analyzed to calculate the localization precision (defined as the standard deviation of the localization error).

### Protocol to coat gold nanoparticles on the coverslip

We first coat the glass bottom dish (FD3510-100, WPI) with poly-D-lysine (P7280, SIGMA) for 20 minutes, followed by 200 µL diluted 100 nm gold nanoparticle solution (1:60 with ddH_2_O, EM.GC100, BBI) for 3 hours. Finally, dishes are coated with another layer of poly-D-lysine for 20 minutes for better cell adherence.

### Cell preparation and staining

Primary mouse embryo fibroblast (MEF) cells or MCF-10A cells were plated onto a PDL (poly-D-lysine) coated glass-bottom dish (FD3510, World Precision Instruments) at confluency of 50% and cultured overnight to let the cells attach to the dish. To stain microtubules, MEF cells were pre-extracted for 30 seconds in 0.5% Triton X-100 (Triton) in BRB80 buffer supplemented with 4 mM EGTA and fixed with Methanol (−20 °C) for 10 minutes. To stain acetylated histone H3, MCF-10A cells were fixed with 4% PFA for 15 minutes and permeabilized with 0.2% Triton for 10 minutes. After fixation, the cells were washed 3 times with PBS and blocked with 3% BSA for 1 hour, then incubated with primary antibody diluted in 3% BSA overnight at 4 °C (rabbit anti-acetyl-histone H3, EMD Milipore 06-599, 1:500). The cells were washed 3 times with PBS and incubated with lab-synthesized Alexa 647-conjugated secondary antibody (Donkey anti rabbit unconjugated antibody, Jackson ImmunoResearch, 711-005-152; Alexa 647 NHS Ester, ThermoFisher, Scientific, A20106) diluted in 3% BSA for 2 hours at room temperature, protected from light. Wash the cells with PBS 3 times and store in PBS until imaging.

Immediately before imaging, the sample was switch to the STORM imaging buffer. For microtubule imaging, buffer contains 10% w/v glucose, 2% v/v β-me, 0.56 mg/mL glucose oxidase, 0.17 mg/mL catalase and 2 mM COT (cyclooctatetraene). For histone imaging, 60% TDE (2,2′-thiodiethanol) was used to dissolve the component above instead of water, and 2 mM COT was added to the buffer solution.

## Electronic supplementary material

Low cost LM supplementary
