# Supplementary material for: A simple and cost-effective setup for super-resolution localization microscopy
Source: Sci Rep. 2017 May 8;7:1542. doi: 10.1038/s41598-017-01606-6 (PMC5431525; doi:10.1038/s41598-017-01606-6)
Supplement: Supplementary file 1 — Low cost LM supplementary [file 41598_2017_1606_MOESM1_ESM.pdf]

A simple and cost-effective setup for super-resolution localization microscopy

Hongqiang Ma [a,1], Rao Fu[1,2], Jianquan Xu[1] and Yang Liu[1,3]

[1]Biomedical and Optical Imaging Laboratory, Departments of Medicine and Bioengineering, University of Pittsburgh, Pittsburgh PA 15213, USA

[2]College of Chemical Engineering, Northeast Electric Power University, China

[3]University of Pittsburgh Cancer Institute, Pittsburgh PA 15213, USA

[a]Corresponding author: hongqiang.ma@pitt.edu

Supplementary Information

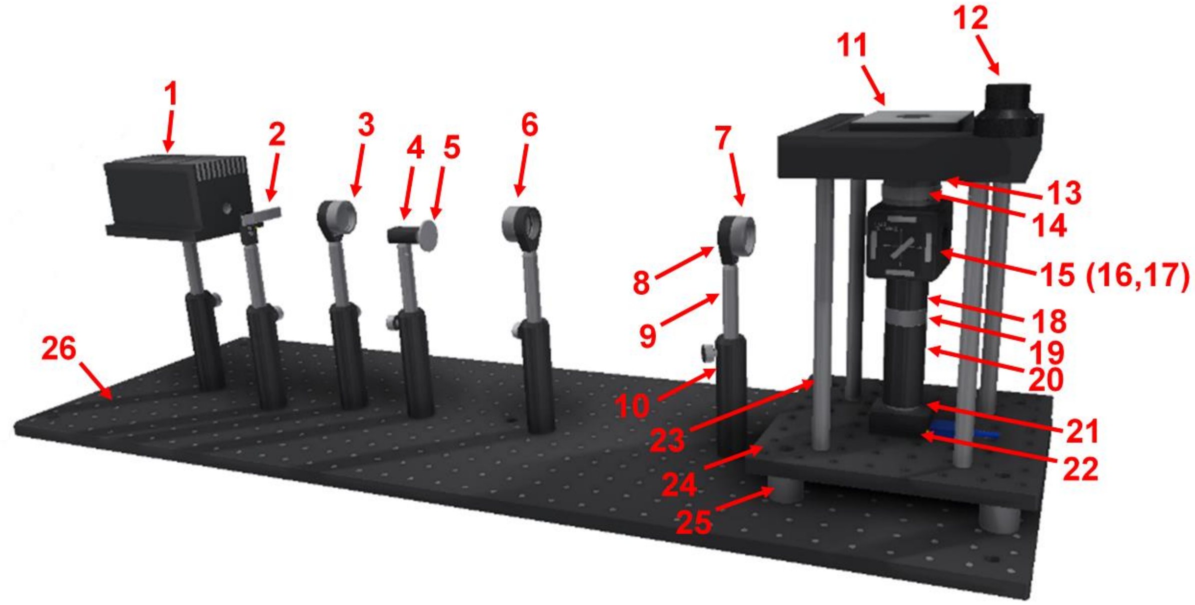

Figure S1. The 3D CAD schematic of the low-cost LM setup and the corresponding detailed list of suppliers and prices for all components is shown in Table S1.

Table S1. Detailed list of major components of the low-cost LM setup.

Module	Item #	Item	Description	Seller (Model #)	Price (USD) X Quantity
Light Source	1	Laser module [a]	Wave length: 638±3 nm, Maximal power: 2000 mW, Output stability: 5%, Beam size: 5 mm, Divergence: <1.5 mrad, M^2: <1.6, TTL/Analog power control	Existotem (item # 331574769076)	$409.0 X1
	2	ND Filter	Rectangular Step ND Filter, 10 mm wide, OD: 0.3 - 2.0	Thorlabs (NDL-10S-2)	$157.00 X1
	3	Lens	f=100 mm, Ø1" Achromatic Doublet, SM1-Threaded Mount, ARC: 400-700 nm	Thorlabs (AC254-100-A-ML)	$95.90 X1
	4	DC motor	6V 250mA Brushed DC Motor, No-load speed: 9100 ±1800 rpm	RobotShop (RB-Ada-142)	$1.95 X1
	5	Engineered diffuser	Round 1 inch, Divergence angle: 5.48º	RPC Photonics (EDC-5-06217-A)	$160.00 X1
	6	Lens	f=100 mm, Ø1" Achromatic Doublet, SM1-Threaded Mount, ARC: 400-700 nm	Thorlabs (AC254-100-A-ML)	$95.90 X1
	7	Lens	f=200 mm, Ø1" Achromatic Doublet, SM1-Threaded Mount, ARC: 400-700 nm	Thorlabs (AC254-200-A-ML)	$95.90 X1
	8	Lens Mount	Lens Mount with Retaining Ring for Ø1" Optics, 8-32 Tap	Thorlabs (LMR1)	$15.23 X3
	9	Optical Post	Ø1/2" Optical Post, SS, 8-32 Setscrew, 1/4"-20 Tap, L = 6"	Thorlabs (TR6)	$6.77 X6
	10	Post Holder	Ø1/2" Post Holder, Spring-Loaded Hex-Locking Thumbscrew, L = 4"	Thorlabs (PH4)	$9.17 X6
Microscope	11	Sample holder [b]	Self-designed sample holder with aperture of 20 mm	Protolabs	$110.00 X1
	12	XY translation stage	X-Y Travel Distances: 75 x 56mm, Active Area: 37.6mm in diameter	AmScope (GT100)	$79.98 X1
	13	Objective lens	100X (Oil) Infinity-corrected Plan Fluor Objective Lens, NA = 1.3, tube length: 160 mm	AmScope (PF100X-INF)	$549.98 X1
	14	Z focusing stage	SM1 Zoom Housing for Ø1" Optics, Non-Rotating, 4.1 mm Travel	Thorlabs (SM1ZM)	$159.10 X1
	15	Filter Cube	Kinematic Fluorescence Filter Cube, 30 mm Cage Compatible, 1/4"-20 Tapped Holes	Thorlabs (DFM1)	$326.00 X1
	16	Dichroic mirror	660 nm edge BrightLine single-edge dichroic beam splitter	Semrock (FF660-Di02-25x36)	$255.00 X1
	17	Emission filter	emission filter for Alexa-647	Chroma (ET700/75m)	$325.00 X1
	18	Spacer	SM1 Lens Tube Spacer, 1" Long	Thorlabs (SM1S10)	$12.00 X1

	19	Tube lens [c]	f=100 mm, Ø1" Achromatic Doublet, SM1-Threaded Mount, ARC: 400-700 nm	Thorlabs (AC254-100-A-ML)	$95.90 X1
	20	Spacer	SM1 Lens Tube, 2.50" Thread Depth, One Retaining Ring Included	Thorlabs (SM1L25)	$20.21 X1
	21	Thread adapter	Adapter with External C-Mount Threads and Internal SM1 Threads	Thorlabs (SM1A9)	$18.75 X1
	22	Camera [d]	Sony IMX265 sensor, 2048 x 1536 3.45 µm pixels, 71% QE, 2.89e- read noise, USB3.0	PointGrey (Chameleon3 CM3-U3-31S4M)	$490.00 X1
	23	Optical Post	Ø1/2" Optical Post, SS, 8-32 Setscrew, 1/4"-20 Tap, L = 8"	Thorlabs (TR8)	$8.12 X4
	24	Microscope base	Aluminum Breadboard 8" x 8" x 1/2", 1/4"-20 Taps	Thorlabs (MB8)	$72.00 X1
	25	Vibration isolating feet	Ø27.0 mm Sorbothane Feet, Internal 1/4"-20 Mounting Thread, 4 Pieces	Thorlabs (AV4)	$22.00 X1
**Optical Table**	26	Breadboard	Aluminum Breadboard 12" x 36" x 1/2", 1/4"-20 Taps	Thorlabs (MB1236)	$313.00 X1
**Total Cost:**	$4038.38				

a: The laser module is a multi-emitter device, which consist of four laser diodes. The output power of this laser module can be controlled by TTL or analog signal. Note that, although there is no visible amplified spontaneous emission (ASE) in the laser module used in this study, some laser modules may require additional excitation filter to block the unwanted ASE, which can create overwhelming background signals.

b: The sample holder is required because the XY translation stage is not designed for imaging samples using small Petri dish. In this work, we machined the customer-designed dish holder in Protolabs. But similar sample holders can be ordered from other companies such as Mad City Labs.

c: Here, we used a tube lens with focal length of 100 mm for proper pixel size (115 nm) on the sample plane. However, this objective lens is originally designed for a tube lens at a focal length of 160 mm. Thus our choice of tube lens increases the spherical aberration.

d: A gain of 25 dB and bin of 2x2 pixels was used in this study. Cameras with IMX250 sensor can also be chosen for better performance.

Table S2. Estimated cost for the major components of the standard LM setup.

Module	Item	Price (USD)
**Light Source** [a]	Single high-power DPSS laser [a] (~$18k) + Shutter (~$2k) + Beam Shaper (~$5k)	~$25k
**Microscope**	Microscope frame (~$8k) + TIRF objective (~$9k)+ Filter set & Drift correction module (~$20k)	~$37k
**Camera** [b]	EMCCD cameras (~35k) / sCMOS cameras (~16k)	~$35k/~$16k
**Optical Table**	Optical Table (~$5k) + Air Compressors (~$1k)	~$6k
**Total Cost :**	~$103k (if EMCCD is used)/~$84k (if sCMOS is used)	

a: The price of the laser source varies with its output power. Here, the price of the laser source is based on a fiber laser with a wavelength of 642nm and output power of 500 mW.

b: Currently, the two mostly used camera in super-resolution localization microscopy is EMCCD camera or sCMOS camera. The price for sCMOS cameras is estimated based on those sold in the US and the price may vary with the specific models, locations and manufacturers.

If users are interested in using SMLM under total internal reflection fluorescence (TIRF) mode, we also evaluated the performance of our setup together with a high-NA TIRF objective, although it is not for low-cost purpose. We found that our method to create the uniform illumination field described in the main text also applies to TIRF imaging. Please note that the full divergence angle of the laser beam in the sample plane is ~3 degree in one dimension and ~12 degree in the other dimension. Therefore, we recommend to position the beam with large divergence angle to be parallel with the lateral plane of the illumination field for TIRF or highly inclined (HILO) illumination. To validate its performance on the optical sectioning under TIRF mode, we used a testing sample in which gold nanoparticles are coated on the surface of the coverslip and MEF cells are grown on top of the gold nanoparticles. Figure S2 below shows a single-frame raw image from sparsely distributed emitters from the fluorescently labeled nuclear lamina located at ~500 nm above the coverslip under STORM imaging condition, in (a) HILO mode and (b) TIRF mode. In the HILO mode, the illumination thickness is over 1 µm, so the fluorophores from nuclear lamina can be excited by the laser (Fig. S2a). In the TIRF mode, these fluorescent signals can be barely observed, and only the gold nanoparticles attached to the coverslip can be excited by the laser (Fig. S2b), which suggests that illumination thickness should be only a few hundreds of nanometers. In summary, the method to generate a uniform beam presented in this study can be applied in epi-illumination, highly inclined illumination and TIRF imaging.

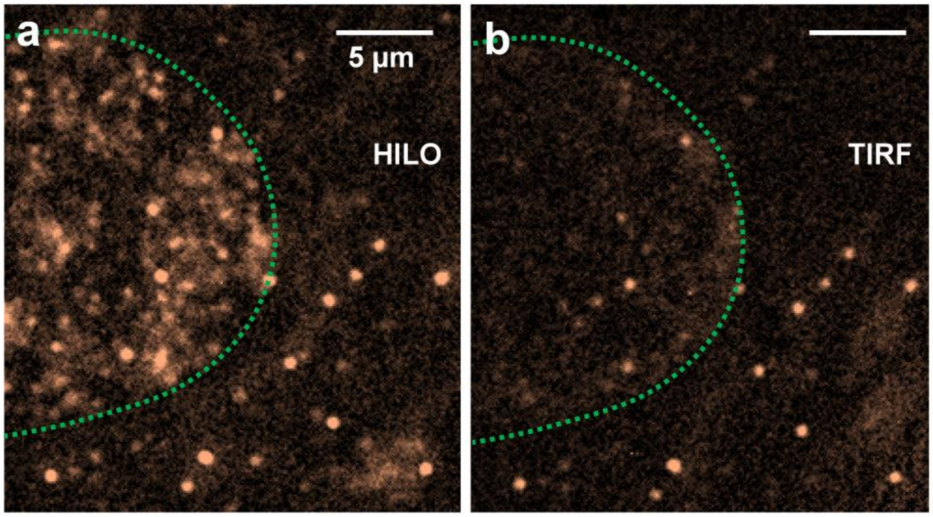

Figure S2. Performance of optical sectioning in our setup equipped with TIRF objective lens (Olympus APON60XOTIRF, NA 1.49) under (a) high inclined illumination (HILO) mode and (b) TIRF mode.

Staining of nuclear lamina proteins: Primary MEF cells were fixed with Methanol: Acetone (1:1) for 10 minutes. Wash the fixed samples 3X with PBS and add blocking buffer (3% BSA + 0.1% Triton X-100 in PBS) to incubate for 1 hour, then add primary anti-rabbit antibody lamin B1 (Abcam, ab1790) at 1:500 concentration and incubate overnight at 4°C. Wash the cells 3X with washing buffer and add the secondary donkey anti-rabbit antibody (Jackson ImmunoResearch, 121165) conjugated with Alexa-647 (ThermoFisher, A20106) in blocking buffer at 1:200 concentration, then incubated for 2 hours at room temperature and protected from light.
